# Non-Invasive Radiofrequency Field Treatment of 4T1 Breast Tumors Induces T-cell Dependent Inflammatory Response

**DOI:** 10.1038/s41598-018-21719-w

**Published:** 2018-02-22

**Authors:** Jared M. Newton, Jose H. Flores-Arredondo, Sarah Suki, Matthew J. Ware, Martyna Krzykawska-Serda, Mahdi Agha, Justin J. Law, Andrew G. Sikora, Steven A. Curley, Stuart J. Corr

**Affiliations:** 1 Baylor College of Medicine, Dept. of Surgery, Houston, TX 77030 USA; 2 Baylor College of Medicine, Dept. of Otolaryngology-Head and Neck Surgery, Houston, TX 77030 USA; 3 Interdepartmental Graduate Program in Translational Biology and Molecular Medicine, Baylor College of Medicine, Houston, Texas 77030 USA; 4 Faculty of Biochemistry, Biophysics and Biotechnology, Jagiellonian University, Krakow, 30-387 Poland; 5 Rice University, Dept. of Mechanical Engineering and Materials Science, Houston, TX USA; 6 Rice University, Dept. of Chemistry & Smalley Institute, Houston, TX 77030 USA; 7 University of Houston, Dept. of Bioengineering, Houston, TX 77004 USA; 8 Swansea University, School of Medicine, Swansea, Wales UK

## Abstract

Previous work using non-invasive radiofrequency field treatment (RFT) in cancer has demonstrated its therapeutic potential as it can increase intratumoral blood perfusion, localization of intravenously delivered drugs, and promote a hyperthermic intratumoral state. Despite the well-known immunologic benefits that febrile hyperthermia can induce, an investigation of how RFT could modulate the intra-tumoral immune microenvironment had not been studied. Thus, using an established 4T1 breast cancer model in immune competent mice, we demonstrate that RFT induces a transient, localized, and T-cell dependent intratumoral inflammatory response. More specifically we show that multi- and singlet-dose RFT promote an increase in tumor volume in immune competent Balb/c mice, which does not occur in athymic nude models. Further leukocyte subset analysis at 24, 48, and 120 hours after a single RFT show a rapid increase in tumoral trafficking of CD4+ and CD8+ T-cells 24 hours post-treatment. Additional serum cytokine analysis reveals an increase in numerous pro-inflammatory cytokines and chemokines associated with enhanced T-cell trafficking. Overall, these data demonstrate that non-invasive RFT could be an effective immunomodulatory strategy in solid tumors, especially for enhancing the tumoral trafficking of lymphocytes, which is currently a major hindrance of numerous cancer immunotherapeutic strategies.

## Introduction

Solid tumor cancers currently make up over 85% of new cancer cases and result in over 90% of all cancer-related deaths in the United States^1^. Despite promising advances in cancer oncology many solid tumors pose a major challenge, often resulting in the use of aggressive treatment schedules, which expose patients to dangerous, potentially deadly, treatment regimens with considerable side-effects. This has lead the field to seek alternative treatment options for solid tumor malignancies and immunotherapy is currently a promising area of investigation. Immunotherapies hold substantial potential as a treatment platform for solid tumors, largely because they provide semi-selective targeting of cancer cells, the ability to attack both local and disseminated disease, and features of immunologic memory, which can recognize and eliminate instances of recurrence. These unique abilities of immunotherapy make it an increasingly attractive treatment strategy in solid tumor therapy.

Despite extensive efforts however, immunotherapies currently only promote long-term survival benefits in a small subset of patients with solid tumor malignancies^2–4^. Many groups propose that various tumor factors are primarily responsible for this lack of efficacy. The first problem is the tumor microenvironment, a highly immunosuppressive environment composed of a variety of regulatory cell types such as myeloid-derived suppressor cells (MDSC) and T-regulatory cells (Treg), which mitigate any potential effector immune responses infiltrating the tumor^5^. Multiple groups have further suggested that tumors possess high intra-tumoral interstitial pressure and high levels of hypoxia due to inadequate blood vascularization which significantly hinders various cells of the immune system from reaching the internal recesses of the tumor^6–8^.

Non-invasive radiofrequency field treatment (RFT) has been previously investigated by our group and others as a potential therapy in both pancreatic ductal adenocarcinoma (PDAC) and hepatocellular carcinoma (HCC)^9–11^. Further characterization of the effects of RFT within *in vitro* systems suggests that RFT induces changes in cell-cell adhesion, elasticity, and morphology, which could significantly change the physical characteristics of the tumor microenvironment^12,13^. Our group has additionally shown that single dose-RFT can enhance intra-tumoral blood flow and perfusion of intravenously delivered nanoparticle probes^14,15^. More recently we demonstrated that consecutive RFT doses across a range of treatment temperatures (37–43 °C) could enhance intravenously delivered fluorescent probes, suggesting that RFT could improve intratumoral drug delivery. Results from this study further suggested that RFT promoted a unique form of tumor hyperthermia, with drastic improvements in temperature differential (i.e. internal tumor temperature vs. systemic body temperature), uniform tumor heating, post-treatment intra-tumoral blood velocity, and overall safety compared to contact-convectively delivered hyperthermia treatment^16^. Thus, RFT proved to be a safe and optimal method for exposing the tumor to hyperthermic conditions.

Hyperthermia has long been a topic of interest in cancer with treatment history dating back to Hippocrates in ancient Greece^17^. Systemic hyperthermia in the febrile range (38 °C –42 °C) has been previously investigated for its chemo- and radiotherapy sensitizing properties, which improved clinical outcomes in a randomized clinical trial^18^. With recent technological developments, a number of studies have investigated more localized hyperthermia effects including isolated limb, intraperitoneal, and thoracic cavity heating, and observed promising enhancements in tumor treatment sensitivity^19,20^. In addition to radio- and chemo-sensitizing properties, hyperthermia is also known to promote a variety of favorable intratumoral immunologic effects^21^. Several potential treatment mechanisms have been suggested, including the ability of febrile range hyperthermia to improve tumor oxygenation: a critical hurdle of immune attack of solid tumors, as hypoxic conditions are known to promote many immunosuppressive effects^8,22^.

In addition to re-oxygenation, numerous studies have suggested that mild hyperthermia can promote effector immune cell tumoral trafficking, with some reported cases showing a greater than 5-fold increase in cytotoxic T cells infiltrating the tumor microenvironment^23^. Although the mechanism of this enhanced infiltration still remains somewhat unclear, prior data would suggest that the mild hyperthermia is able to drive various features which favor lymphocyte infiltration and attack of solid tumors; including increasing expression of vessel wall binding domains such as intercellular adhesion molecule-1 (ICAM-1)^24^, promoting a more favorable intratumoral interstitial pressure^25^, and driving the production of a number of pro-inflammatory cytokines and chemokines (i.e. interleukin (IL)−1β, IL-6, IL-8, IL-10) which have major implications in T-cell activation and trafficking^26,27^. Despite the vast amount of work done to investigate intratumoral hyperthermia immunologic effects, most studies rely on either systemic or convectively delivered hyperthermia. Based on our prior data, we have demonstrated that RFT can provide more localized, uniform, and safe intratumoral hyperthermia; however, an investigation of immunologic effects following febrile range RFT hyperthermia remains unstudied.

Therefore, the objective of this work was to characterize the immunologic changes induced by RFT with a particular focus on intratumoral immune microenvironment changes. We hypothesized that RFT would induce significant intra-tumoral immune changes particularly through pro-inflammatory mechanisms. Using immune competent Balb/c mice bearing 4T1 breast tumors we investigated both consecutive RFT dosing schedules and single-dose time-course investigations to better understand the transient immunologic changes induced by RFT.

## Results and Discussion

**Figure 1:**
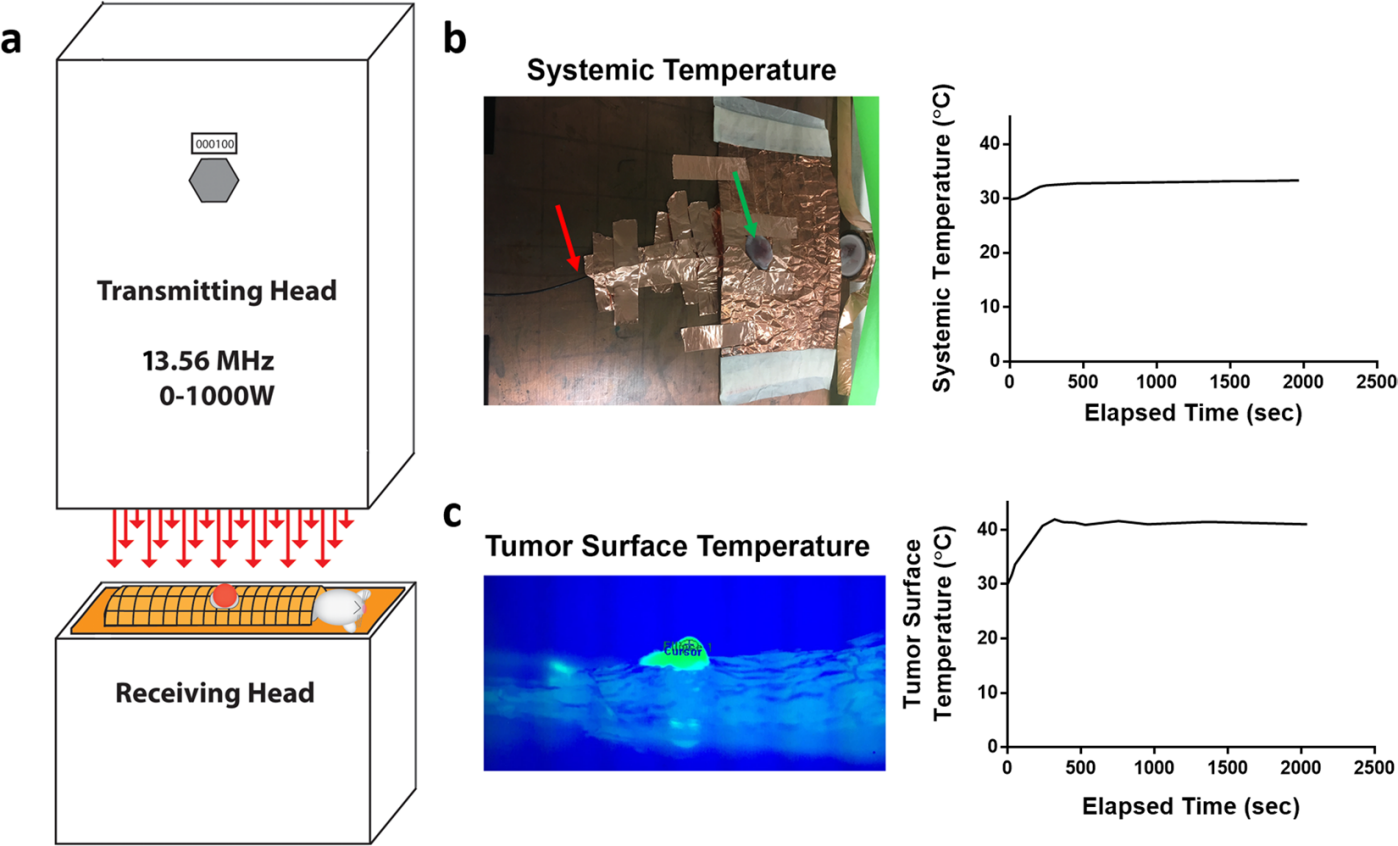
RFT set-up and temperature monitoring. (**a**) Schematic depicting capacitively-coupled radiofrequency (RF) transmitting and receiving head showing mouse orientation and copper blanket shielding. (**b**) Image of mouse grounding and shielding showing exposed tumor (green arrow) and rectally inserted fiber-optic probe (red arrow) used for systemic temperature monitoring. Representative graph to the right shows systemic temperature measurement for a single mouse during an entire RFT session. (**c**) Image from infrared camera showing exposed tumor used for tumor surface temperature monitoring. Representative graph to the right shows tumor surface temperature measurement for a single mouse during an entire RFT session. (Supplemental Figure S1 shows cumulative treatment systemic and tumor surface heating curves).

Using the protocol developed in our previous study^16^, either Balb/c or athymic nude Balb/c mice bearing 4T1 tumors were treated with multiple doses of RFT. During treatment, tumor surface temperatures were increased to 41 °C and maintained there for 30 mins and systemic temperature of each mouse was measured using a rectally inserted fiber optic temperature probe. Using this method allowed for effective and consistent heating of tumor tissue and only minor systemic heating (Fig. 1a–c; Supplementary Figure S1A). Based on previous published work this would be consistent with a 40–41 °C internal tumor temperature, as RFT results in a less than 1 °C temperature differential between superficial and intra-tumoral temperature^16^. Control treated mice were placed on a heating pad under isoflurane to maintain similar systemic temperatures as the RFT mice, to better investigate the localized heating effects.

**Figure 2:**
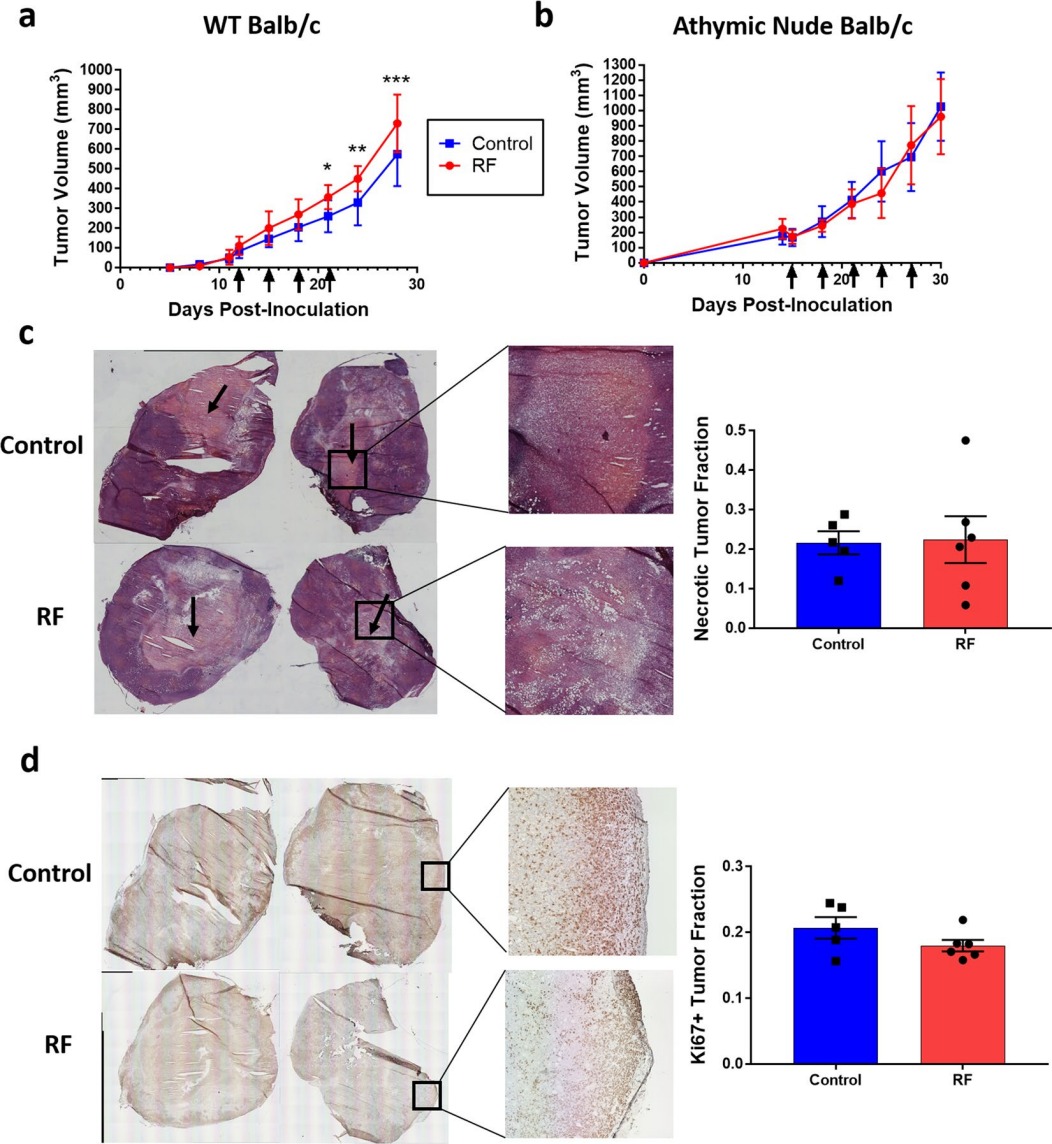
Consecutive-dose RFT induces T-cell dependent tumor growth effect with no effects on tumor necrosis or proliferation. 4T1 tumor volume following multiple consecutive RFT (41 °C, 30 mins; date of treatment indicated by black arrows) in either (**a**) wild-type Balb/c mice or (**b**) athymic nude Balb/c mice (n = 10/group). (**c**) Representative H&E histology images of control (top) and RF-treated (bottom) tumors at termination (black arrow denotes necrotic region), with zoom-in of necrotic region to the right. Quantification of necrotic fraction is provided to the far right comparing RF-treated and control mice (n = 5–6/group). (**d**) Representative IHC images showing Ki67 expression comparison between control (top) and RF-treated (bottom) tumors, with zoom-in of highly proliferative tumor periphery to the right. Quantification of Ki67+ tumor fraction is provided to the far right comparing RF-treated and control mice (n = 5–6/group). Error bars represent SEM. (See Supplemental Figure S3 for complete image set). (*p < 0.05, **p < 0.01, ***p < 0.001).

RFT or control mice, either Balb/c or athymic nude Balb/c, bearing established 4T1 tumors underwent numerous consecutive treatments every 3 days and tumor growth was measured throughout. Of interest, the RFT Balb/c mice demonstrated a significant increase in tumor volume compared to control Balb/c mice after 4 total radiofrequency (RF) doses (Fig. 2a). This increase in tumor volume was not observed in the immunodeficient athymic nude Balb/c mice bearing similarly established 4T1 tumors following multiple RFT (Fig. 2b). Histologic analysis of these tumors revealed no major changes between the RF and control treated mice and quantification of tumor necrotic fraction revealed similar necrosis between both groups (Fig. 2c). In addition, Ki67 staining for tumoral proliferation showed similar levels between control and RF treated groups (Fig. 2d; see Supplementary Figure S3A,B for full set of images). Both control and RF treated groups showed strong proliferation in the outer tumor periphery as expected per this aggressive 4T1 tumor model (Fig. 2d). Furthermore, although only mouse body weight was directly assessed, there were no obvious differences between RFT and control groups to suggest treatment-related toxicities (Supplementary Figure S1). In addition, we noted no signs of metastases at termination, and similar lung weights between RF and control mice further suggest no increase in metastatic potential following RFT (Supplementary Figure S1C). Collectively, these data suggest that RFT induced an inflammatory response within tumors that required functional T-cells and that this immunologic effect was primarily responsible for the change in tumor volume after multiple doses of RFT.

**Figure 3:**
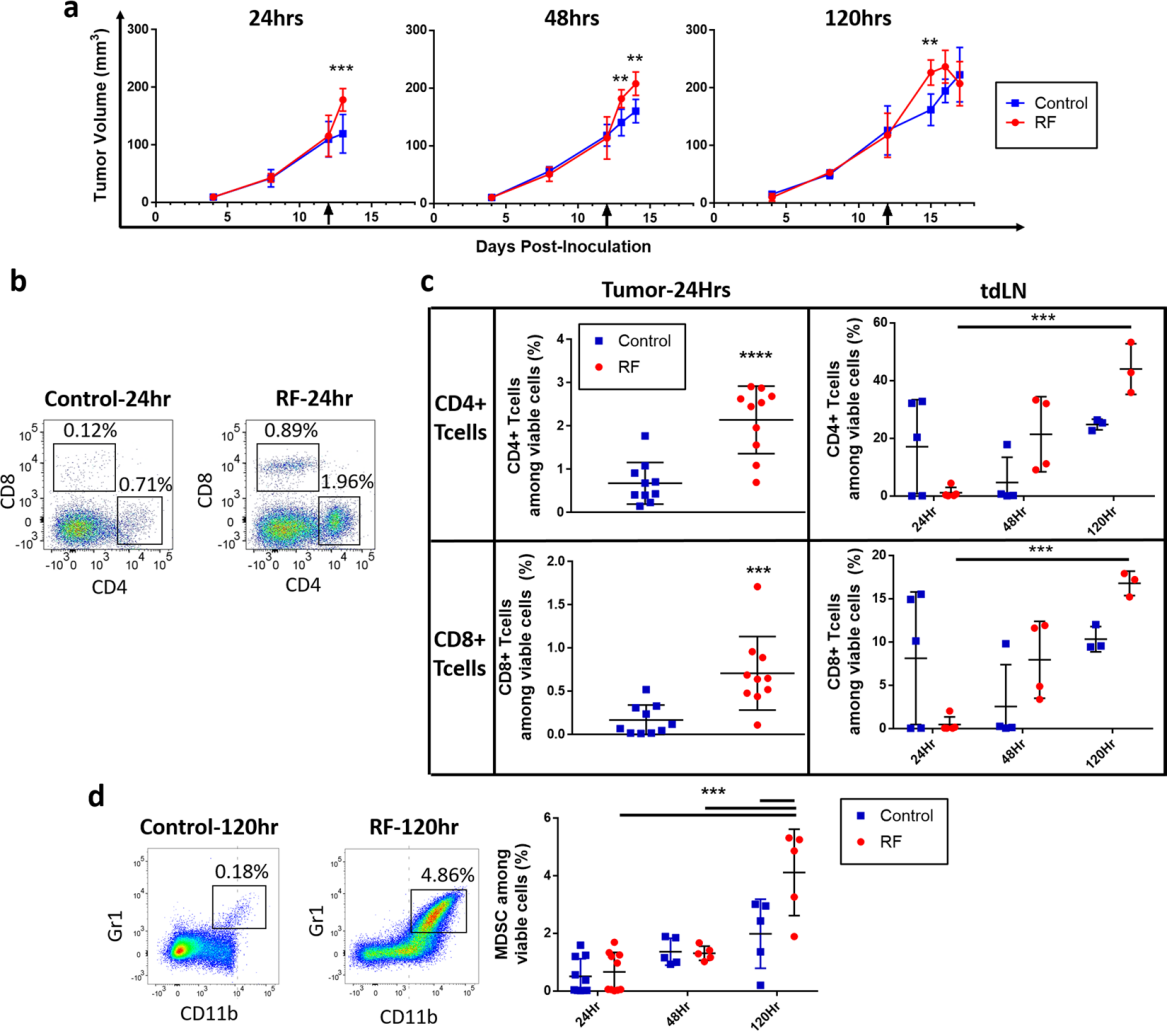
Immune microenvironment time-course analysis following single-dose RFT. (**a**) 4T1 tumor volume in Balb/c mice following single RFT (41 °C, 30 mins; day of treatment indicated by black arrow) which were terminated for microenvironment analysis at either 24hrs (left), 48hrs (middle), or 120hrs (right) post-RFT (n = 5/group). (**b**) Representative flow cytometry scatter plots among CD45+ cells showing the increase in CD4+ and CD8+ T cell populations in tumor between a control and RF-treated mice 24 hours post-RFT. (**c**) Cumulative plots of CD4+ T cell percentages (top row) and CD8+ T cell percentages (bottom row) showing changes in T cell percentages among total viable cells 24hrs post-treatment for tumor (left column) and changes 24, 48, and 120hrs post-treatment in tumor-draining lymph node (right column). (n = 3–10/group). (**d**) Control vs. RF-treated tumor percentage of MDSC (CD11b+/Gr1+) 120hrs post-treatment with representative flow plots (left) and cumulative plots showing percentages among total viable cells (right) (n = 5–10/group). See Supplemental Figs S4 and S5 for flow cytometry gating strategy. Each point represents an individual mouse percentage and all percentages are out of total viable cells analzyed. Error bars represent SEM. (*p < 0.05, **p < 0.01, ***p < 0.001, ****p < 0.0001).

To better characterize the immunologic changes induced by RFT, an immune microenvironment analysis of tumor, spleen, blood, and tumor-draining inguinal lymph node was performed (see Supplemental Figs S4 and S5 for flow gating strategy). For these studies immune competent Balb/c mice with established 4T1 tumors were treated with a single dose of RFT. Tissues were then processed and analyzed either 24, 48, or 120 hours after single-dose RFT. Similar to multi-RFT tumor growth characteristics, single-dose RFT tumors also displayed a transient tumor volume increase. RFT treated tumors showed a 50% increase in measured volume by 24 hours post-RFT which remained significantly larger than control tumors until 72 hours post-RFT, after which they returned to control tumor sizes (Fig. 3a). Immune microenvironment analysis further showed a transient tumoral influx of CD4+ and CD8+ T-cells which were increased by greater than 3- and 4-fold in RFT treated tumors 24 hours after treatment, respectively (Fig. 3b,c). Increased T-cell levels returned to control levels by 48 hours after RFT. Analysis of tumor-draining inguinal lymph nodes showed an inverse effect as they appear entirely devoid of T-cells 24 hours post-RFT, but significantly increase more than 38- and 36-fold for CD4+ and CD8+ T-cells, respectively, by 120 hours post-RFT (Fig. 3c). These data suggest that lymph-node dwelling T-cells are being drawn to the tumor rapidly after RFT is applied, rendering the lymph node devoid of lymphocytes. In addition, it would appear that the increases in T-cell trafficking following RFT are time-dependent, potentially peaking near 24 hours post-RFT. One potential factor that could be limiting further lymphocyte trafficking into tumors that received RFT is the 2-fold increase in MDSC at 120 hours post-RFT compared to control tumors (Fig. 3d). MDSC are well-known to employ a number of immunosuppressive mechanisms that can significantly inhibit the infiltration of lymphocytes into the tumor microenvironment^5^. Thus, the increased MDSC may contribute to the build-up of T-cells within the lymph node as they inhibit further T-cell infiltration to the tumor microenvironment.

Splenic and hematologic changes appeared quite minor with only slight decreases in CD4+ T-cells, CD8+ T-cells, and macrophages in the blood at 24 hours post-RFT, which could be explained by the observed trafficking and other tumoral effects of RFT (Supplementary Tables 1–4). In addition, the activation state of tumor dwelling CD8+ T-cells was also significantly reduced at 48 hours post-RFT, as they expressed lower levels of Perforin and IFNγ, two T-cell effector molecules (Supplementary Figure S2). The immunologic effects of RFT appear to be highly localized to the site of treatment, with only minor systemic alterations. Therapeutically this could provide an advantage to many other immunomodulatory treatments, which have concerning systemic toxicities, usually involving systemic hyperactivation and concerns of cytokine storm. Overall, our data would suggest that RFT can provide a highly localized and transient influx of lymphocytes which, if combined with other lymphocyte-focused immunotherapeutic strategies, will likely provide major combinatorial therapeutic benefit.

**Figure 4:**
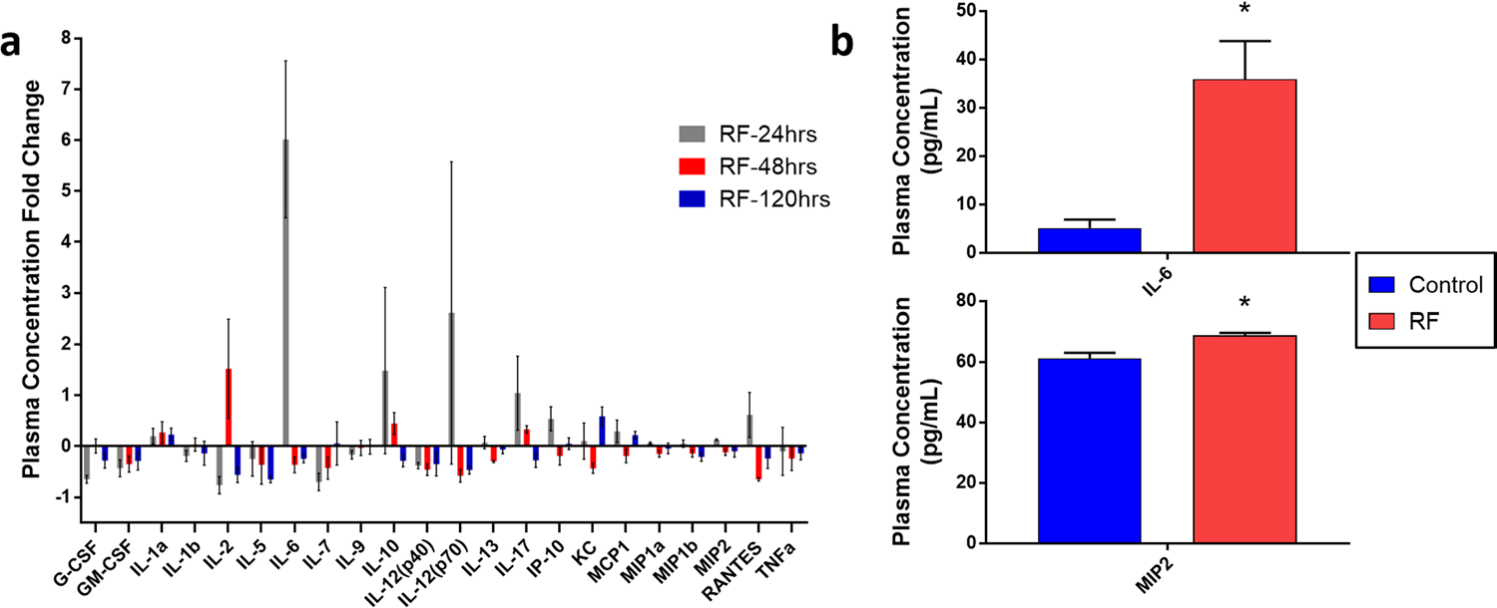
Blood plasma cytokine panel time-course analysis following single-dose RFT. At 24, 48, or 120 hrs after a single RFT blood plasma was analyzed for 25 cytokines. (**a**) Cytokine plasma concentration fold-change of RF-treated mice at 24, 48, or 120 hours post-RFT compared to control mice. Fold changes for each analyte were calculated by taking the ratio of average plasma concentrations of RFT-mice and control mice for each independent time-point (i.e. [G-CSF]_24 hr RF_/[G-CSF]_24 hr Control_) (n = 5/group). (**b**) Plasma concentration in pg/mL of IL-6 (top) and MIP2 (bottom) comparing control and RF-treated mice 24hrs post-RFT (n = 5/group). Error bars represent SEM. (*p < 0.05).

Due to the vast amount of previous evidence suggesting that key pro-inflammatory cytokine and chemokine expression following febrile hyperthermia promote T-cell infiltration^26,27^, we investigated whether RFT induced any significant changes in serum cytokine levels. Blood collected at 24, 48, and 120 hours after a single RFT were compared across a pro-inflammatory panel of 25 cytokines and chemokines. Similar to previous results we observed a highly transient cytokine response, with noted increases 24 hours after RFT in a number of proinflammatory cytokines including IL-6, IL-17, IP-10, IL-12(p70), MIP2, and RANTES (Fig. 4a). IL-6 appeared the most drastically elevated, with a 6-fold increase induced 24-hours after a single dose of RFT (Fig. 4b). Despite the negative effects that IL-6 is known to induce in tumors, previous evidence would suggest that IL-6 alters its function under period of hyperthermic stress and strongly promotes T-cell activation and enhanced infiltration^26^.

While modest, a significant increase was also noted in MIP2 (i.e. CXCL2), a potent chemoattractant for leukocytes (Fig. 4b). This along with the other modest increases in chemokines such as RANTES (i.e. CXCL5) and IP-10 (i.e. CXCL10) could collectively contribute to the enhanced infiltration of T-cells by 24-hours post-RFT. On the other hand, G-CSF, a cytokine commonly released by endothelium and macrophages, underwent a 2.5-fold reduction 24-hours after RFT. This observation was further supported by the microenvironment analysis which showed that intra-tumoral macrophage were almost entirely depleted 24 hours post-RFT (Supplementary Figure S2). Since this depletion was not associated with increased trafficking to the draining lymph node, this would suggest that tumor dwelling macrophages may have a lower tolerance for hyperthermia conditions resulting in their depletion. Interestingly, all elevated cytokines and chemokines appear to return to baseline levels by 48 hours post-RFT, with the exception of IL-2, which modestly increases at 48 hours post-RFT. The transient nature of the cytokine and chemokines directly corroborates our microenvironment analysis, which showed the most potent intratumoral infiltration effects at 24 hours post-RFT. Overall, these data further suggest a transient immunologic effect induced by RFT, and suggest that the enhanced T-cell tumoral infiltration could be due at least in part by increased expression of a number of proinflammatory cytokines and leukocyte attractive chemokines.

## Conclusion

With the emergence of immunotherapy as a promising cancer treatment modality there remains an evident need for immunomodulatory strategies capable of promoting effector immune cell trafficking and infiltration into solid tumors. The use of non-invasive RFT, despite previously having been shown to promote effective and safe intratumoral hyperthermia, has never been fully investigated for its immunomodulatory potential. Herein we described the immunomodulatory effects of RFT on a 4T1 murine breast tumor model. We observed that RFT-induced hyperthermia drives a highly localized and transient intra-tumoral inflammatory response. This effect promoted a 3- and 4-fold increase in the intra-tumoral infiltration of CD4+ and CD8+ T-cells, respectively, 24 hours after a single RFT was applied. This effect was further manifested as a transient tumor volume increase, which remained significantly larger than control tumors up to 72 hours after a single RFT was applied.

The transient increase in tumor volume that was noted following either single or multiple dose RFT could be associated with previous reports of febrile range hyperthermia in solid tumors that have noted similar transient tumoral swelling occurring shortly after treatment; however, a proper assessment of causation was not performed^28,29^. In addition, numerous reports have suggested that febrile hyperthermia can increase intratumoral blood perfusion, which could cause increased fluid accumulation and tumor distention^30^. It has also been suggested that hyperthermia treatment of cancer cells can promote increased rates of DNA damage^31,32^, which could contribute to delayed tumor volume reduction. All of these reports suggest likely contributing factors to tumor volume enlargement following hyperthermia, however, our data would suggest that local T-cell infiltration plays a contributing role. As support of this claim, we observed significant increases in both CD4+ and CD8+ T-cells within the same duration as the noted tumor enlargement. In addition, the increase in tumor size was not observed in similarly treated athymic nude Balb/c mice, which suggests that functional T-cells are a necessary component of the tumor enlargement phenomenon in this 4T1 murine tumor model. Finally, the observation that neither tumor proliferation or tumor necrosis increased following multiple dose RFT compared to control tumors further suggests that the enlargement was independent of the tumor cell features themselves. Thus, our data suggests that T-cell infiltration and induced inflammation could contribute to transient increases in tumor volume following RFT hyperthermia of solid tumors.

We further provide some potential mechanistic insights for the increased lymphocytes trafficking as various cytokines associated with inflammation and T-cell trafficking were elevated in RFT mice, especially IL-6. Despite these promising effects however, treatment efficacy of RFT alone was not observed, even after multiple consecutive doses. This limitation is likely related to numerous factors including; 1) the lack of tumor-specific and effector T-cell generation, 2) increased trafficking of immunosuppressive MDSC populations after RFT, 3) T-cell exhaustion or lack of critical lymphocyte signaling, and 4) persistent expression of immunosuppressive molecules by malignant cells. This highlights the need to further combine RFT with other immunotherapeutic strategies, which could promote lymphocyte activation, enhance T-cell tumor specificity, and prevent exhaustion; such as immune checkpoint inhibitors, chimeric antigen receptor (CAR) T-cells, and cancer vaccine strategies.

Overall, these data demonstrate that non-invasive RFT may provide an effective transient immunomodulatory method in solid tumor cancers, and its combination with other immunotherapeutic strategies will likely provide significant combinatorial therapeutic benefit. Future experimental work will focus on repeated RFT to maintain higher levels of effector T cells in the tumor microenvironment and on studying improved immunotherapy-based cancer cell cytotoxicity while co-treating with checkpoint inhibitors and other immunomodulators.

## Materials and Methods

### Ethic statement and general mice conditions

All experiments were performed with approval of the Institutional Animal Care and Use Committee (IACUC) of Baylor College of Medicine (No. AN-6445) and followed established protocols. Female Balb/c or athymic nude Balb/c mice (Jackson Labs) were housed in standard temperature and lighting conditions with free access to food and water. RFT was performed under isoflurane anesthesia (0.7–2.5% isoflurane in medical air). During anesthesia, systemic mouse temperature was monitored using a rectally inserted fiber optic temperature probe and breathing frequency was maintained at approximately 1 Hz by adjusting isoflurane concentration and/or flow rate. After anesthesia and treatment mice were kept in a pre-warmed chamber until complete recovery.

### Tumor model

4T1 cells were purchased from American Type Cell Culture (ATCC; Rockville, MD) and were cultured in RPMI 1640 media supplemented with 10% fetal bovine serum (FBS) and 1% penicillin/streptomycin. Cells were cultured in a humidified atmosphere at 37 °C and 5% CO_2_. 10^5^ 4T1 breast cancer cells suspended in base medium were injected into the left inguinal gland (27 G needle, 50 µL injection volume) to initiate orthotopic 4T1 breast tumors in Balb/c mice (wildtype or athymic nude). Treatment (RF or control) was initiated between 12–15 days of tumor development when tumors had reached 100–200 mm^3^ in volume. All tumor measurements were performed using calipers and volume was calculated using Equation 1, where W = tumor width, L = tumor length, and H = tumor height.

1 $$V=\frac{\pi }{6}\ast L\ast W\ast H$$

### Radiofrequency field treatment (RFT)

For a single dose of RFT anesthetized mice were grounded and shielded using copper tape to ensure localized RFT at the tumor site. Mice were then subjected to high intensity (~90 kV/m) 13.56 MHz RF fields at various powers (0–1000 W) to administer a bi-phasic thermal dose that included a ‘ramp up’ phase from baseline tumor surface temperature to 41 °C and a second ‘plateau’ phase which maintained tumor surface temperature at 41 °C for 30 mins. Non-RF “control” mice were similarly anesthetized and placed on a heating pad to achieve similar levels of systemic heating without the addition of RFT. Systemic temperature and tumor surface temperature were measured using a rectally inserted fiber optic thermal probe and an infrared camera, respectively (for treatment set up see Fig. 1a–c). For both control and RFT mice, the skin on and around the tumor were shaved prior to treatment to allow for accurate tumor surface temperature assessment throughout treatment. All inoculated mice were randomized prior to treatment and the tumor measurements were performed with proper blinding. Before experiments were performed, the IR camera and fiber optic probes were calibrated using a water-bath (data not shown) to ensure accurate thermometry.

### Multiple dose RFT

Consecutive doses of RFT or control treatment were performed in established 4T1 breast tumors in Balb/c mice (wildtype or athymic nude) as described above and in our previous work^16^. After tumors reached size requirements treatment was initiated and repeated every 3 days for a total of 4–5 doses. Growth assessment was terminated between day 28–30 post-tumor inoculation.

#### Tissue Sectioning and Histology

At termination, tumors were isolated, frozen in O.C.T (Tissue-Tek), and stored at −80 °C prior to histologic sectioning and staining. Frozen sections were cut at −20 °C at 6 µm thickness and picked up on positively charged slides. Slides undergoing hematoxylin and eosin (H&E) staining were fixed in 95% ethyl alcohol for 2 minutes prior to standard H&E staining. Unstained slides were stored at −80 °C until further processing.

#### Ki67 immunohistochemistry

Slides were fixed in chilled acetone for 20 minute prior to staining. Heat-induced epitope retrieval was performed using a Biocare Decloaking chamber and Rodent Decloaking solution at 125 °C for 30 minutes (Biocare Medical, LLC). Slides then underwent primary Ki67 antibody incubation for 30 minutes, MACH2 universal detection incubation for 30 minutes, and Betazoid DAB chromogen for 5 minutes, with thorough washing using Tris-buffered saline between each incubation. After the final rinse, slides were background stained using CAT hematoxylin for 5 minutes. Fully stained slides were thoroughly rinsed and dehydrated using an ethanol gradient series prior to coverslipping.

#### Microscopic imaging of histology

All brightfield imaging was performed using a Nikon Eclipse TE2000-U microscope fitted with a Nikon digital sight DS-Fi1 camera. Image analysis was performed using ImageJ software (National Institutes of Health, USA).

### Single dose RFT Time-course

A single dose of RFT or control treatment was delivered at day 12 post-tumor inoculation and leukocyte subset analyses of tumor, spleen, blood, and tumor draining inguinal lymph node was performed at 24, 48, or 120 hrs after the single treatment.

#### Tissue preparation

At termination, tumor, spleen, blood, and tumor draining inguinal lymph node was harvested. Tumor was manually chopped into pieces and digested in base media containing 1 mg/mL Collagenase I (Sigma) for 1 hr at 37 °C with gentle shaking throughout. At 1 hr, digestions were immediately ceased with the addition of media containing 2% FBS. Digested tumors, spleens, and lymph nodes were passed through a 30 µm cell strainer to obtain single cell suspensions. Blood was collected via cardiac puncture and split between a tube containing EDTA to prevent clotting (for microenvironment analysis) and a free tube, which was allowed to clot at room temperature (for serum cytokine collection). Single cell suspension of spleen and EDTA-containing blood underwent red blood cell lysis using LCK lysis buffer per manufacturer’s instructions. Clotted blood from the free tube was centrifuged (2000 × g, 10 mins) and supernatant serum was collected and stored at −80 °C prior to analysis.

#### Staining and Flow cytometry

Single cell suspensions for microenvironment analysis were stained for flow cytometric analysis using either a lymphocyte or myeloid staining panel. Antibodies for the lymphocyte panel are as follows; CD45 APC-eFluor780, CD4 PE-Cy5, CD8a eFluor450, NK1.1 AF700, PD-1 PerCP-eFluor710, CTLA-4 PE, IFNγ PE-eFluor610, Granzyme B eFluor660, Perforin FITC, and FoxP3 PE-Cy7. Antibodies for the myeloid panel are as follows; CD45 APC-eFluor780, F4/80 eFluor450, CD11b APC, PD-L1 PE-Cy7, iNOS FITC, CD11c PE-Cy5.5, MHCII PE, Ly6G (Gr1) AF700. All antibodies were purchased from Ebiosciences except iNOS which was purchased from BD Bioscience. Both panels optimized a viability stain, FVS510 (BD Biosciences). For staining, cells were first stained with viability stain, rinsed, and blocked in Fc Block (BD Bioscience) per manufacturer’s instructions. Extracellular staining was performed in 100 µL total staining volume protected from light for 30 mins at 4 °C. Cells were then rinsed and fixed/permeabilized (Fix/Perm buffer eBioscience) at 4 °C overnight. The following day cells were rinsed and resuspended in Perm buffer (eBioscience), stained for intracellular markers (30 mins at 4 °C), rinsed, and resuspended in FACs buffer (PBS + 2% FBS) for flow cytometry analysis using an LSR II flow cytometer (BD Bioscience). Flow cytometry analysis was performed using FlowJo v10 (FlowJo, LLC; Ashland, OR). For all cell types analyzed percentages were recorded as percent of total viable cells analyzed because a similar number of total viable cells were recorded between each sample (see Supplemental Figs S4 and S5 for gating strategy for lymphocyte and myeloid panels, respectively).

#### Serum Cytokine Analysis

Serum cytokine analysis optimized a 25-plex mouse cytokine/chemokine magnetic bead panel (EMD Millipore). Cytokines/chemokines assayed include G-CSF, GM-CSF, IFNγ, IL-1α, IL-1β, IL-2, IL-4, IL-5, IL-6, IL-7, IL-9, IL-10, IL-12 (p40), IL-12 (p70), IL-13, IL-15, IL-17, IP-10, KC, MCP-1, MIP-1α, MIP-1β, MIP-2, RANTES, and TNF-α. Samples were prepared via manufacturer’s instructions and analysis was performed using a Luminex LX200 (Luminex Corp. Austin, TX).

### Experimental design and data analysis

All experiments were done with proper blinding and randomization. For all experiments GraphPad Prism version 7.00 for Windows (La Jolla, CA) was used for graph generation and statistical analysis. Normal data distribution was verified by Shapiro-Wilk test prior to comparisons. Bi-comparisons (i.e. RFT vs. control) were statistically compared using an unpaired student’s t-test. Multi-comparisons (i.e. tumor growth curves, serum cytokine analysis) were performed using a 2-way ANOVA with Bonferroni-corrected multi-comparison. For all comparisons, a p-value less than 0.05 was considered statistically significant and the following p-value representations were used throughout; *p < 0.05, **p < 0.01, ***p < 0.001, ****p < 0.0001.

### Data Availability

The datasets generated during and/or analyzed during the current study are largely included in this published article (and its Supplementary Information files) and any other datasets generated not included in this article are available from the corresponding author on reasonable request.

## Electronic supplementary material

Supporting Information
