# Supplementary material for: Non-Invasive Radiofrequency Field Treatment of 4T1 Breast Tumors Induces T-cell Dependent Inflammatory Response
Source: Sci Rep. 2018 Feb 22;8:3474. doi: 10.1038/s41598-018-21719-w (PMC5823899; doi:10.1038/s41598-018-21719-w)
Supplement: Supplementary file 1 — Supporting Information [file 41598_2018_21719_MOESM1_ESM.pdf]

Non-Invasive Radiofrequency Field Treatment of 4T1 Breast Tumors Induce T-cell Dependent Inflammatory Response

Jared M. Newton, Jose Flores-Arredondo, Sarah Suki, Matthew J. Ware, Martyna Krzykawska-Serda, Mahdi Agha, Justin J. Law, Andrew G. Sikora, Steven A. Curley, and Stuart J. Corr

Supplementary Information

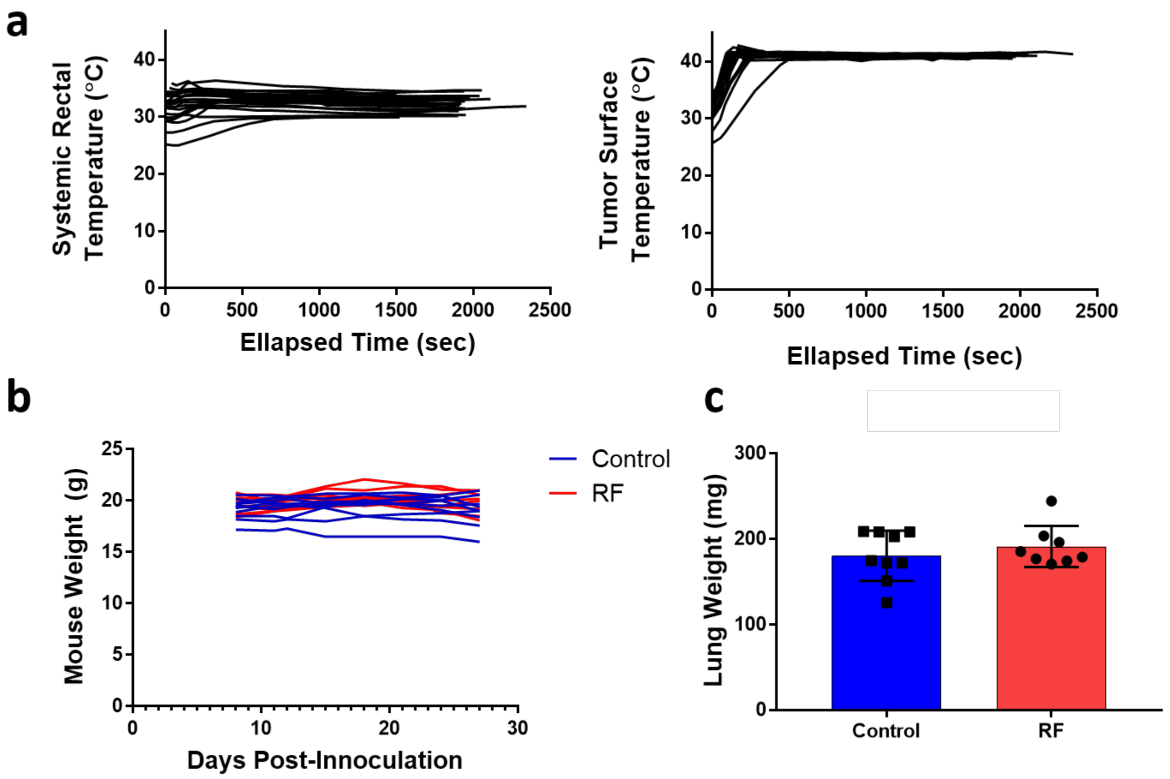

Figure S1: RFT promotes consistent and safe intratumoral hyperthermia. a) Representative systemic temperature measurements from rectally inserted fiber-optic probe (left) and tumor surface temperature measurement from IR camera for RFT mice bearing 4T1 tumors showing changes during an entire treatment duration (n=10 mice, 2 different treatment days). **b**) Mouse weight changes in grams following multiple treatments between RFT and control-treated mice (n=10/group; for schedule see Figure 2A). **c**) Mouse lung weights (mg) after multiple treatments between RFT and control-treated mice (n=10/group; each dot represents an individual mouse lung weight).

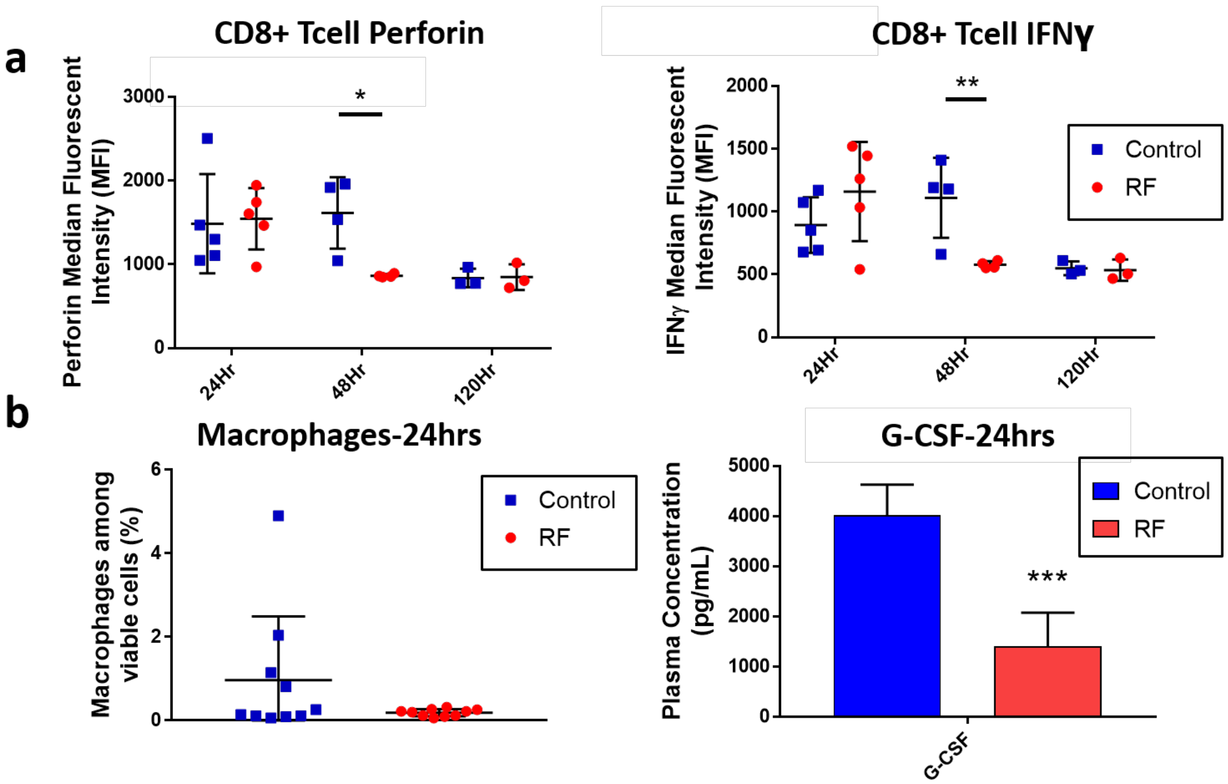

Figure S2: RFT induced changes in T-cell activation and macrophages. a) Cumulative dot plots showing perforin (left) and IFNγ (right) median fluorescent intensity among tumor dwelling CD8+ T-cell at 24, 48, and 120 hours post-treatment(n=5/group). **b**) Tumor macrophage percentages among total viable cells at 24, 48, and 120 hours post-treatment (left; n=5/group). Plasma concentration in pg/mL of G-CSF comparing control and RF-treated mice 24hrs post-RFT (right; n=5/group)

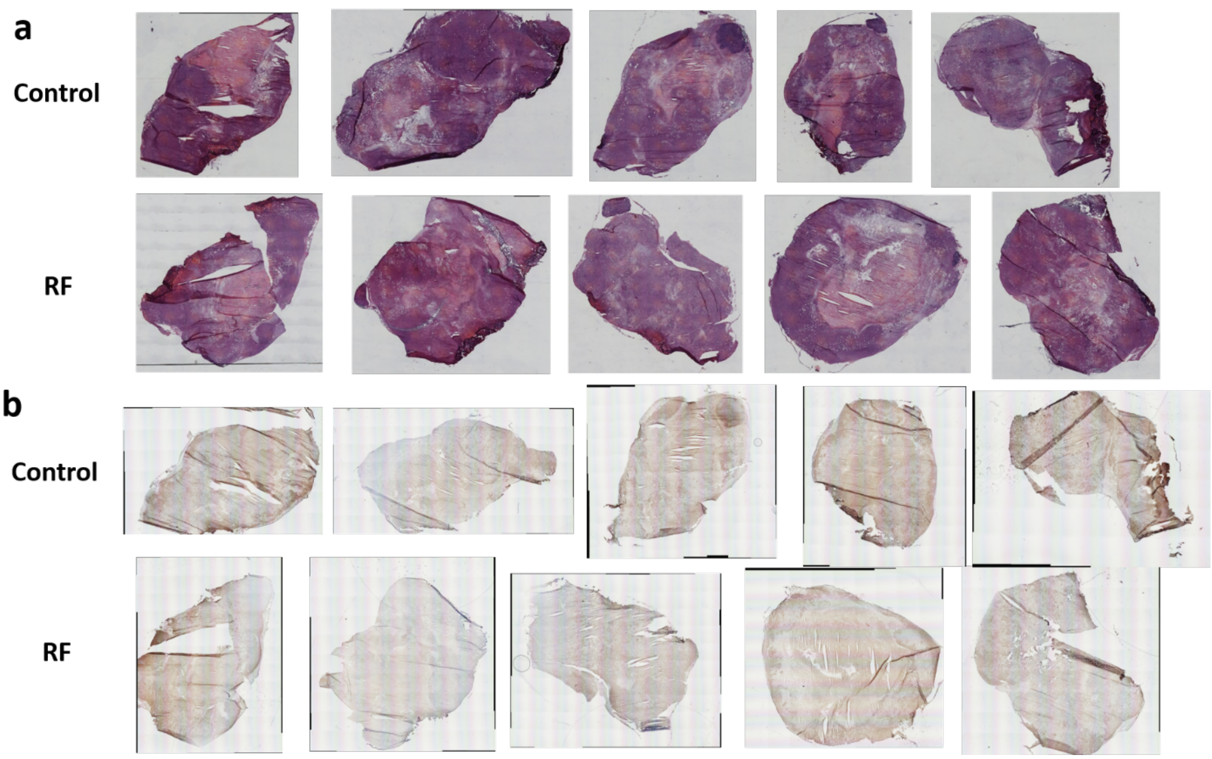

Figure S3: H&E and Ki67 histology of control and RFT tumors. Full image set showing **a**) H&E staining and **b**) Ki67 immunohistochemical staining following multiple treatments, either RFT or control (see Figure 2a for treatment schedule).

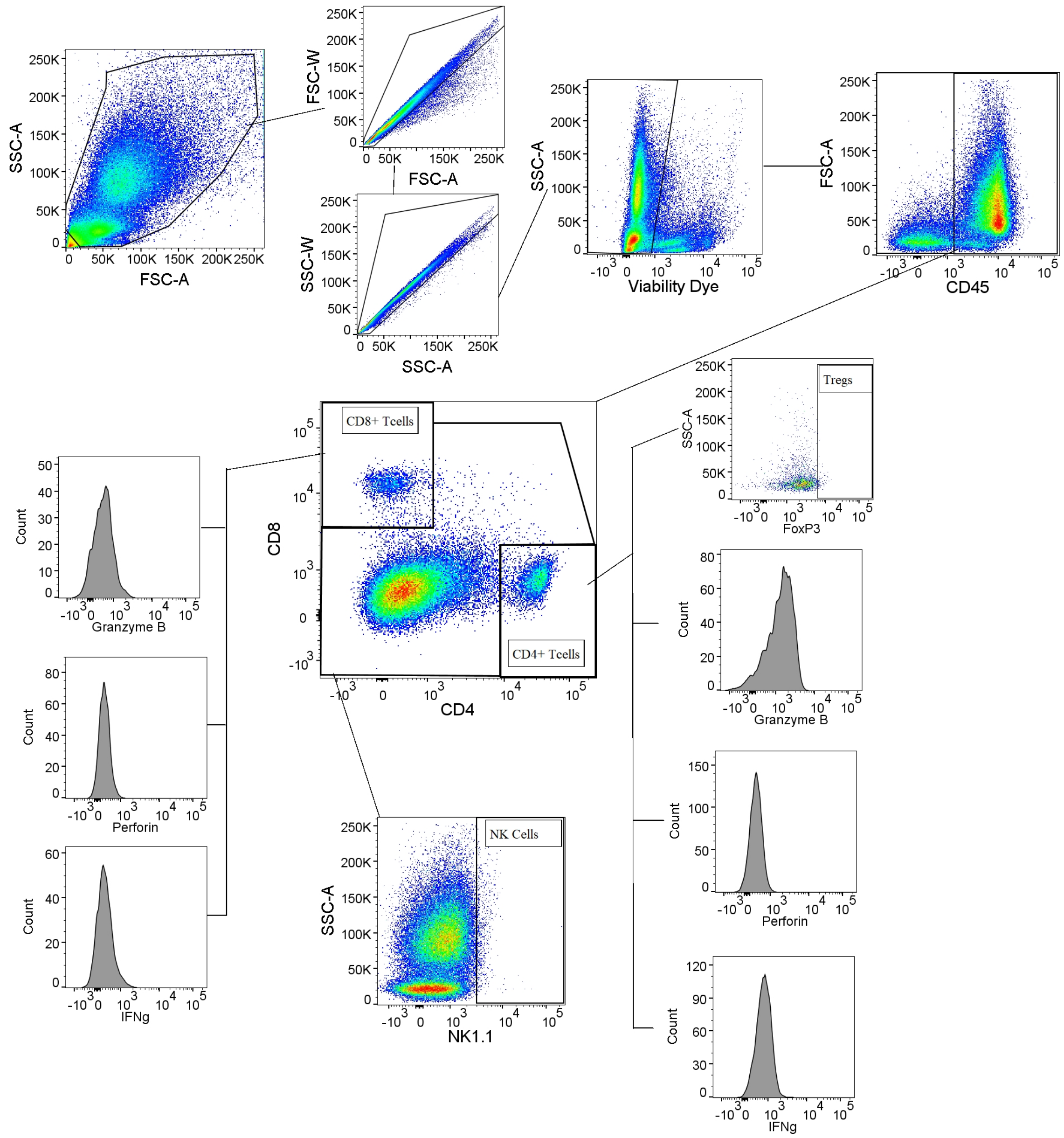

Figure S4: Immune microenvironment lymphocyte-focused flow cytometry gating panel. Representative flow cytometry plots and histograms showing gating strategy used for quantification of lymphocyte cell types and features in the immune microenvironment study. All cell types were represented as a percentage of total viable cells analyzed.

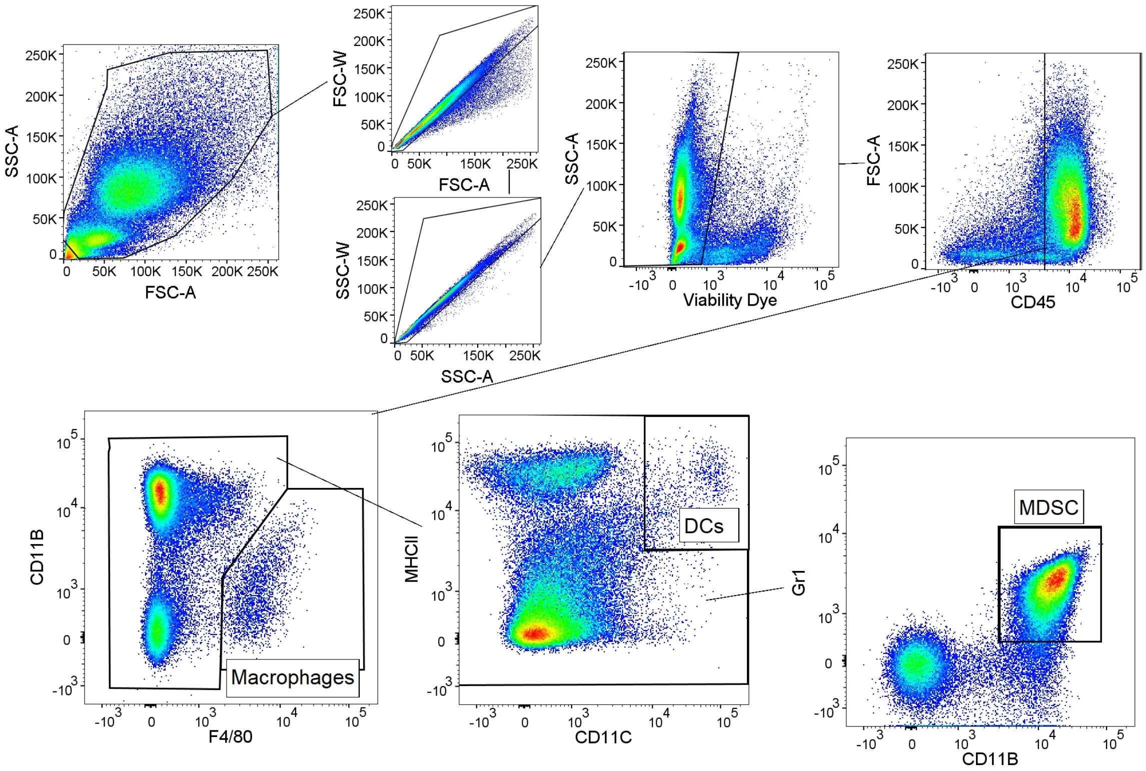

Figure S5: Immune microenvironment myeloid-focused flow cytometry gating panel. Representative flow cytometry plots showing gating strategy used for quantification of myeloid cell types in the immune microenvironment study. All cell types were represented as a percentage of total viable cells analyzed.

Supplementary Table 1: Changes in primary immune cell subsets in the tumor, all as a percent of total viable cells. Data presented as average (SEM).

	Tumor-24hrs		Tumor-48hrs		Tumor-120hrs	
	Control %	RF %	Control %	RF %	Control %	RF %
CD4+ Tcell (CD45+/CD4+)	0.677 (0.152)	2.141 (0.246)	1.042 (0.586)	0.420 (0.136)	0.954 (0.339)	0.435 (0.222)
CD8+ Tcells (CD45+/CD8+)	0.169 (0.054)	0.709 (0.134)	0.248 (0.147)	0.057 (0.033)	0.204 (0.107)	0.098 (0.052)
Treg (CD45+/CD4+/FoxP3+)	0.129 (0.040)	0.230 (0.085)	0.646 (0.471)	0.215 (0.085)	0.5782 (0.220)	0.2742 (0.163)
NK Cell (CD45+/NK1.1+)	0.005 (0.001)	0.005 (0.001)	0.004 (0.002)	0.002 (0.001)	0.002 (0.001)	0.001 (0.001)
Macrophage (CD45+, F4/80+, CD11b^{Intermediate})	0.968 (0.482)	0.189 (0.027)	3.032 (1.356)	2.206 (1.022)	4.384 (0.449)	4.742 (0.555)
DC (CD45+, F4/80-, CD11c+, MHCII^{High})	1.874 (0.193)	1.394 (0.235)	1.892 (0.127)	1.394 (0.198)	1.936 (0.510)	2.652 (0.423)
MDSC (CD45+, F4/80-, CD11c-, CD11b+, Gr1+)	0.512 (0.193)	0.666 (0.215)	1.366 (0.209)	1.312 (0.110)	0.996 (0.225)	1.288 (0.117)

Supplementary Table 2: Changes in primary immune cell subsets in the spleen, all as a percent of total viable cells. Data presented as average (SEM).

	Spleen-24hrs		Spleen-48hrs		Spleen-120hrs	
	Control %	RF %	Control %	RF %	Control %	RF %
CD4+ Tcell (CD45+/CD4+)	4.620 (0.907)	5.703 (1.583)	4.238 (1.406)	4.344 (0.289)	4.380 (0.780)	5.158 (0.766)
CD8+ Tcells (CD45+/CD8+)	1.869 (0.500)	2.107 (0.683)	1.840 (0.596)	1.448 (0.145)	2.206 (0.410)	2.416 (0.377)
Treg (CD45+/CD4+/FoxP3+)	0.148 (0.051)	0.220 (0.080)	0.095 (0.037)	0.210 (0.022)	0.159 (0.036)	0.244 (0.071)
NK Cell (CD45+/NK1.1+)	0.280 (0.091)	0.251 (0.084)	0.700 (0.128)	0.856 (0.079)	0.438 (0.062)	0.538 (0.082)
Macrophage (CD45+, F4/80+, CD11b^{Intermediate})	2.536 (0.698)	2.838 (0.691)	3.898 (0.495)	4.422 (0.439)	2.996 (0.418)	2.896 (0.276)
DC (CD45+, F4/80-, CD11c+, MHCII^{High})	1.304 (0.106)	1.312 (0.068)	1.260 (0.200)	1.44 (0.079)	0.860 (0.241)	1.050 (0.094)
MDSC (CD45+, F4/80-, CD11c-, CD11b+, Gr1+)	20.014 (6.775)	16.584 (6.030)	46.54 (4.766)	43.12 (2.541)	35.980 (9.932)	43.200 (3.455)

Supplementary Table 3: Changes in primary immune cell subsets in the tumor draining inguinal lymph node, all as a percent of total viable cells. Data presented as average (SEM).

	tdLN-24hrs		tdLN-48hrs		tdLN-120hrs	
	Control %	RF %	Control %	RF %	Control %	RF %
CD4+ Tcell (CD45+/CD4+)	17.067 (7.287)	1.149 (0.824)	4.68 (4.374)	21.398 (6.516)	24.767 (1.073)	44.000 (5.059)
CD8+ Tcells (CD45+/CD8+)	8.115 (3.428)	0.462 (0.390)	2.545 (2.415)	7.937 (2.223)	10.32 (0.841)	16.767 (0.809)
Treg (CD45+/CD4+/FoxP3+)	2.707 (1.510)	0.123 (0.085)	0.638 (0.584)	1.220 (0.380)	3.033 (0.462)	2.660 (0.535)
NK Cell (CD45+/NK1.1+)	0.085 (0.021)	0.299 (0.127)	0.258 (0.084)	0.058 (0.013)	0.068 (0.042)	0.057 (0.021)
Macrophage (CD45+, F4/80+, CD11b^{Intermediate})	0.080 (0.034)	0.588 (0.321)	0.059 (0.025)	0.043 (0.008)	0.033 (0.009)	0.019 (0.006)
DC (CD45+, F4/80-, CD11c+, MHCII^{High})	0.601 (0.264)	0.394 (0.197)	0.191 (0.140)	0.288 (0.046)	0.270 (0.099)	0.323 (0.131)
MDSC (CD45+, F4/80-, CD11c-, CD11b+, Gr1+)	0.126 (0.027)	0.492 (0.154)	0.578 (0.207)	0.064 (0.003)	0.189 (0.076)	0.160 (0.036)

Supplementary Table 4: Changes in primary immune cell subsets in the blood, all as a percent of total viable cells. Data presented as average (SEM).

	Blood-24hrs		Blood-48hrs		Blood-120hrs	
	Control %	RF %	Control %	RF %	Control %	RF %
CD4+ Tcell (CD45+/CD4+)	1.454 (0.498)	0.752 (0.139)	0.682 (0.159)	0.988 (0.106)	1.168 (0.186)	1.012 (0.115)
CD8+ Tcells (CD45+/CD8+)	0.502 (0.175)	0.250 (0.050)	0.206 (0.048)	0.300 (0.029)	0.416 (0.061)	0.376 (0.039)
Treg (CD45+/CD4+/FoxP3+)	0.304 (0.079)	0.286 (0.199)	0.290 (0.164)	0.180 (0.097)	0.854 (0.190)	0.560 (0.159)
NK Cell (CD45+/NK1.1+)	0.026 (0.017)	0.007 (0.002)	0.005 (0.002)	0.006 (0.001)	0.023 (0.007)	0.075 (0.033)
Macrophage (CD45+, F4/80+, CD11b^{Intermediate})	0.538 (0.216)	0.193 (0.062)	0.242 (0.056)	0.226 (0.028)	1.900 (1.413)	0.632 (0.340)
DC (CD45+, F4/80-, CD11c+, MHCII^{High})	0.017 (0.005)	0.009 (0.001)	0.015 (0.003)	0.014 (0.002)	0.012 (0.004)	0.016 (0.003)
MDSC (CD45+, F4/80-, CD11c-, CD11b+, Gr1+)	15.26 (6.839)	6.55 (0.870)	11.230 (1.828)	7.808 (1.089)	10.580 (3.256)	7.216 (1.962)
